# Systematic Review of Cancer Targeting by Nanoparticles Revealed a Global Association between Accumulation in Tumors and Spleen

**DOI:** 10.3390/ijms222313011

**Published:** 2021-12-01

**Authors:** Andrey S. Drozdov, Petr I. Nikitin, Julian M. Rozenberg

**Affiliations:** 1 Laboratory of Nanobiotechnology, Moscow Institute of Physics and Technology, 141700 Dolgoprudny, Russia; drozdov.science@gmail.com; 2 Prokhorov General Physics Institute of the Russian Academy of Sciences, 119991 Moscow, Russia; petr.nikitin@nsc.gpi.ru; 3 Cell Signaling Regulation Laboratory, Moscow Institute of Physics and Technology, 141700 Dolgoprudny, Russia

**Keywords:** nanoparticles for drug delivery, functionalization, cancer targeting, nanoparticle therapy, biodistribution

## Abstract

Active targeting of nanoparticles toward tumors is one of the most rapidly developing topics in nanomedicine. Typically, this strategy involves the addition of cancer-targeting biomolecules to nanoparticles, and studies on this topic have mainly focused on the localization of such formulations in tumors. Here, the analysis of the factors determining efficient nanoparticle targeting and therapy, various parameters such as types of targeting molecules, nanoparticle type, size, zeta potential, dose, and the circulation time are given. In addition, the important aspects such as how active targeting of nanoparticles alters biodistribution and how non-specific organ uptake influences tumor accumulation of the targeted nanoformulations are discussed. The analysis reveals that an increase in tumor accumulation of targeted nanoparticles is accompanied by a decrease in their uptake by the spleen. There is no association between targeting-induced changes of nanoparticle concentrations in tumors and other organs. The correlation between uptake in tumors and depletion in the spleen is significant for mice with intact immune systems in contrast to nude mice. Noticeably, modulation of splenic and tumor accumulation depends on the targeting molecules and nanoparticle type. The median survival increases with the targeting-induced nanoparticle accumulation in tumors; moreover, combinatorial targeting of nanoparticle drugs demonstrates higher treatment efficiencies. Results of the comprehensive analysis show optimal strategies to enhance the efficiency of actively targeted nanoparticle-based medicines.

## 1. Introduction

Targeted delivery of drugs is important for the safety and efficiency of cancer treatment. A common approach to increase the specificity of drug delivery is to encapsulate them into nanoparticles that preferentially accumulate in tumor tissues due to either enhanced permeability and retention (EPR) effect, the controversial concept that the increased leakiness of the tumor vasculature and poor lymphatic drainage can lead to intratumoral accumulation and retention of nanoformulations [1], or due to the decoration of the nanoparticles with antibodies or ligands that specifically bind to their targets, and thus, are overexpressed or presented exclusively in the tumor vasculature or cells [2,3,4]. In some cases, the EPR effect can be responsible for up to 32% of the nanoparticle injected dose delivered to selected tumors [5,6,7]. However, one of the specific reasons for slow progress in nanomedicine development is that the EPR effect observed in mice cancer models [1,8] is not as profound or not working at all in human cancers [3,9,10,11,12]. Moreover, most recent studies showed that the importance of the EPR effect might be overestimated for the intratumoral accumulation of nanoformulations in animal models [13,14]. Therefore, several strategies have been developed to enhance or bypass the requirements for the EPR effect for drug delivery [3,12]. Alternative methods to enhance the efficiency of nanoparticle drug delivery to tumors by so-called “targeting” remain to be the subjects of thousands of investigations [2,3,4,15,16].

Examples of ligand tumor-specific molecule binding pairs include small molecules: folic acid (FA) binding to the folic acid receptor overexpressed in breast and many other cancers [17,18], anisamide binding to the sigma receptor overexpressed by tumor-associated fibroblasts [19,20], binding of tetraiodothyroacetic acid to the thyroid hormone receptor in renal cell carcinoma and breast cancer [21,22], and to a ligand of the prostatic specific membrane antigen (PSMA) for prostate cancer [23]. An example of polymer binding is hyaluronic acid (HA) that binds to the CD44 expressed by the cancer stem cells [24,25]. Other examples include antibodies or their fragments that bind to the variety of cancer- and cancer microenvironment-associated proteins (EGFR, HER2, PD-1, CD8, PD-L1, VEGFR, etc.) [26,27,28,29,30], and peptides, such as internalizing iRGD [31,32] that binds to the integrins and is internalized by the neuropilin-1 overexpressed by the endothelium of different cancers, or the non-internalizing RGD peptide [33,34] that binds to integrins. Aptamers designed to bind several targets [35,36] including nucleolin [37,38] or EPCAM [39], overexpressed in breast cancer, or aptamers selected to bind to breast cancer 4T1 cells [40], are also of relevance. It should be noted that the affinity of recognition molecules immobilized on nanoparticles for target receptors can be four orders of magnitude higher than those for molecular entities due to polyvalent interactions [41,42].

Tremendous efforts over the last half-century to develop nanoparticles for drug delivery led to eight approved drugs for cancer treatment and 11 new nanoparticle formulations in clinical trials [43]. All the approved drugs are derivatives of liposomes, albumin-based paclitaxel nanoparticles (Abraxane), or a micelle form of paclitaxel (Apealea). Clinically approved nanoparticles have fewer side effects than soluble forms of drugs, and, consequently, higher drug concentrations can be used, leading to improvements in progression-free survival [43]. Moreover, nanoparticles targeted by the transferrin receptor (NCT02354547), anti-human epidermis growth factor antibodies (NCT01702129, NCT02369198, NCT02766699), a cRGDY peptide interacting with integrins (NCT02106598), and iRGD—another integrin-interacting peptide with tumor-penetrating ability (NCT03517176)—are in clinical trials (Table 1).

One drawback of the targeting approaches is that they can also change interactions of nanoparticles with healthy cells, thus affecting accumulation in organs, where they can cause side effects [44,45,46,47]. The vast majority of the nanoparticle drugs are captured by the Kupffer macrophages and excreted by the hepatobiliary system [5,48,49,50,51,52,53] or spleen macrophages [54,55,56,57], thereby lowering bioavailability and tumor uptake [58,59]. The kidneys preferentially excrete proteins and nanomaterials of the sub-6 nm size. Many papers report the biodistribution of the targeted and non-targeted nanoparticles, as well as the kinetics of the nanoparticle biodistribution [17,28,33,34,60,61,62,63,64,65,66,67,68,69,70,71,72]. How changes of nanoparticle sequestration by organs caused by the targeting molecules’ influence on tumor accumulation is not understood.

We hypothesized that cancer-targeting-induced changes in nanoparticle biodistribution to organs influence nanoparticle accumulation in tumors. In this work, we systematically characterized nanoparticle targeting research and asked a question: are there any approaches that have the best cancer targeting, and how does this correlate with the changes of biodistribution profiles, and is it translated into better cancer treatment?

## 2. Results and Discussion

### 2.1. Physiological Mechanisms of Cancer-Specific Nanoparticle Accumulation

Cancer metabolism and microenvironments are different from those of normal tissue. These differences include changes in pH acidification and higher lactate concentration due to the Warburg effect: the preferential use of lactate-generating glycolysis even in the presence of oxygen [73]. Moreover, inside cancer cells, there is a reversal of pH gradient, i.e., cells become more basic inside, while tumors become more acidic outside the cells [74]. Cancer cells express a plethora of immunosuppressing molecules, including soluble adenosine, TGF-b, Il2, and IL10 [75]. Tumor cells remodel their microenvironment and extracellular matrix structure via overexpression of the metalloproteinases [76,77]. Typically, cancers recruit other cells to support their growth and repress the immune system including cancer-associated fibroblasts, microphages, myeloid derives suppressor cells, and Treg lymphocytes [78,79]. All these changes can be explored for the specific targeting of cancer vs. normal tissues by targeting both intracellular and extracellular targets [4,29,77,80,81,82,83,84,85,86].

To achieve accumulation in the tumors, nanoparticles should have a sufficiently long circulation time to have a chance to reach tumor vasculature and, possibly, penetrate into the tumor. This is supported by the preferential tumor accumulation observed upon the increasing of the nanoparticle circulation time [5,64,87,88].

Several papers address the question of how nanoparticles penetrate into tumor tissue. For example, transferrin-conjugated, PEGylated gold nanoparticles accumulate in tumors at a higher rate and quantity than their non-targeted counterparts [89]. Whereas non-targeted particles penetrate into the tissue up to 15 µm for 15 nm particles, 8 µm for 30 nm, and 4 µm for 60 nm particles, the tissue penetration of the targeted particles was even lower, suggesting that the ligand coating prevents diffusion into the tissue [89]. These numbers suggest that particles do not penetrate deeper than a single cell volume. Indeed, particles mostly accumulate around vessel walls and do not penetrate into the tumor tissue even though the transferrin is transported through the endothelial cells [90]. Moreover, the same research group found that only a small fraction of particles decorated by FA or Her2 antibodies penetrate into tumors, and only a small fraction is internalized into the cells, the majority of which are vessel-proximal macrophages [91]. Therefore, the passive diffusion of nanoparticles into the cancer tissue is ineffective in the cancer microenvironment.

To transport molecules such as lipids, the neuro-mediators, small RNA, or oxygen nature relies on specific carriers such as lipoproteins, exosomes, or red blood cells that are organized to ensure the specific delivery of “goods” to their cellular targets. The specificity of the delivery is determined by the receptors on the cell membranes that interact with specific molecules on the “carrier” to mediate endocytosis. Many tumor-specific nanoparticle delivery systems employ transcytosis, i.e., active cellular transport across physiological barriers [31,32,70,72,90,92,93,94,95,96,97,98,99,100,101,102,103]. These include the targeting of nanoparticles and free drugs by the tumor-penetrating peptide iRGD, which mediates binding to αv-integrins on the tumor endothelium, and a proteolytic cleavage then exposes a binding motif for neuropilin-1, which mediates cellular endo- and transcytosis of the carrier and penetration into the cells and tissue [31,32,70,72,92,93,99,100,101,102,103,104].

The second well-known molecule used for active cellular transport is the transferrin receptor. Normally, the transferrin receptor is involved in the internalization and recycling of the transferrin, carrying Fe^3+^ via clathrin-mediated caveolae formation, where Fe^3+^ is released at lower pH in the endosome and the free transferrin is recycled to the cells’ surfaces. The transferrin receptor on the endothelial cells mediates transcytosis—the transfer of the transferrin from basolateral to the apical side of the blood–brain barrier [90]. The transferrin receptor is overexpressed not only on the brain endothelium but also by many cancer cells [105] including glioblastoma [106], breast [107], prostate [108], colorectal cancer [109], hepatocellular carcinoma [110], and non-small cell lung cancer [111]. Several nanocarriers designed for the penetration of the blood–brain barrier and the cancer treatment use transferrin or anti-transferrin receptor antibodies [89,106,107,112,113,114,115,116].

Another example of successful use of the transcytosis for nanoparticle traffic is albumin transfer mediated by the interaction with the albumin receptor Gp60 [94,95,96,97,98]. Interestingly, native albumin is transferred via binding to Gp60 and SPARC, whereas maleic anhydride, modified or absorbed to gold nanoparticles of albumin, binds to gp30 and gp18 [94,95]. It appears that modified albumin is not transported across bovine lung microvascular endothelial cell monolayers [94], suggesting that native albumin is preferred for nanoparticle preparations. The albumin receptors Gp60 and SPARC are overexpressed on the surface of cancer cells and cancer endothelium, and cancers use albumin as a source of amino acids [96,117,118]. Many nanoparticle formulations use albumin for building and targeting blocks [97,119,120,121,122,123,124,125]. The albumin-based nanoparticle drug Abraxane is used for metastatic breast cancer and clinical trials for other cancers are underway [97].

Several transcytosis-based strategies resulted in excellent tumor specificity but were not included in the analysis because “non-targeted” nanoparticles are not available, for example, for nanoparticles made of the targeting protein itself [126]. These also include transferrin nanoparticles [127], albumin nanoparticles [128], or albumin nanoparticles modified with transferrin [122].

One of the other important targeting strategies relies on the interactions of the nanoformulations with specific cells migrating to tumors. These are exemplified by the targeting of the immune cells traveling to cancers [29,129], including specific subpopulations of monocytes [130,131] and interactions with tumor-associated macrophages [72,129,132,133]. Mesenchymal stem cells are known to migrate to tumors over a long distance, and their ability to deliver nanoparticles has been thoroughly investigated [134,135,136]. Another example is the preferential homing of erythrocytes carrying nanoparticles to the lung metastasis [137]. Intriguingly, single-walled carbon nanotubes are almost exclusively taken up by a single immune cell subset, Ly-6C(hi) monocytes, almost 100% of Ly-6C(hi) cells uptake nanotubes, and 20% of the nanotubes in the tumor are associated with the Ly-6C(hi) monocytes [130]. A number of studies have focused on the mechanisms of nanoparticle interactions with the immune system [4,138,139,140], macrophage-mediated particle uptake [55,58,141], and microphage-nanoparticle-targeting [72,91,129,132,133,142,143]. Interestingly, nanoparticles can accumulate in the tumor-associated macrophages, which serve as a local drug depot, from which a DNA-damaging particle payload is gradually released to neighboring tumor cells [142].

While for different nanoformulations one of the described mechanisms can predominate, it should be noted that for a specific nanoparticle formulation and cancer type, a combination of these events might determine their pharmacokinetics and tumor accumulation.

### 2.2. Nanoparticle Cancer Targeting Efficiency Correlates with Changes in Spleen Accumulation Mechanisms of Cancer-Specific Nanoparticle Accumulation

**Figure 1:**
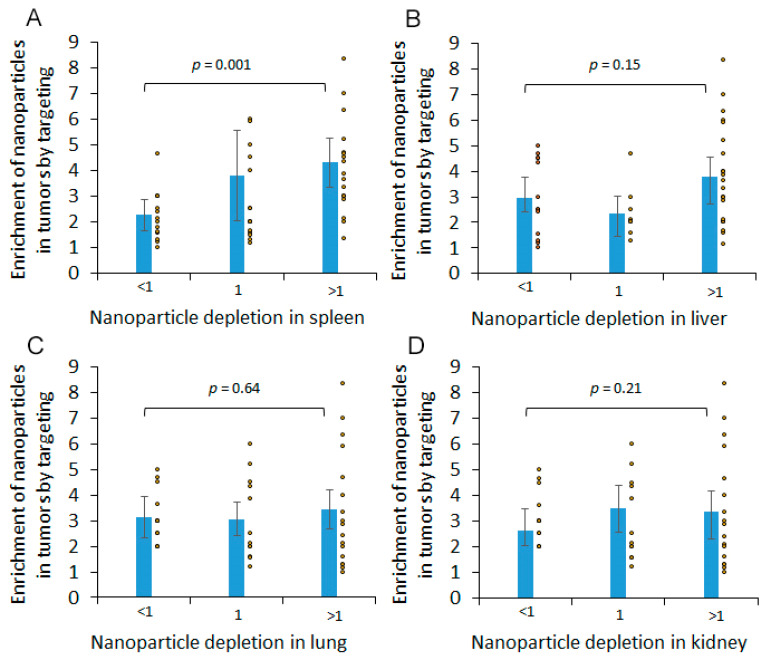
Nanoparticle targeting and biodistribution parameters. Targeting-induced enrichment of nanoparticles in (**A**) spleen, (**B**) liver, (**C**) lungs, and (**D**) kidney. Dots represent individual *ENT* values and bars represent 95% confidence intervals.

Targeting molecules not only change nanoparticle concentrations in tumors but can also change their accumulation in normal tissue. In turn, reduction in the nanoparticle sequestration by the liver and spleen can increase their bioavailability and tumor accumulation. We hypothesized that changes in the nanoparticle biodistribution caused by the cancer-specific molecules influence their accumulation in tumors. To address this question, we compared the enrichment of nanoparticles in tumors (*ENT*) induced by the targeting molecules and the corresponding depletion of the nanoparticles in organs (DR), calculated according to Equations (1) and (2) in the Materials and Methods section. There was a significant difference between the average *ENT* values for the nanoparticles enriched and depleted in the spleen (*p* = 0.0015) (Figure 1). For lung, kidney, liver, and heart, the average values for nanoparticles enriched and depleted in the organs were not different (Figure 1, and the data are not shown for the heart). 

**Figure 2:**
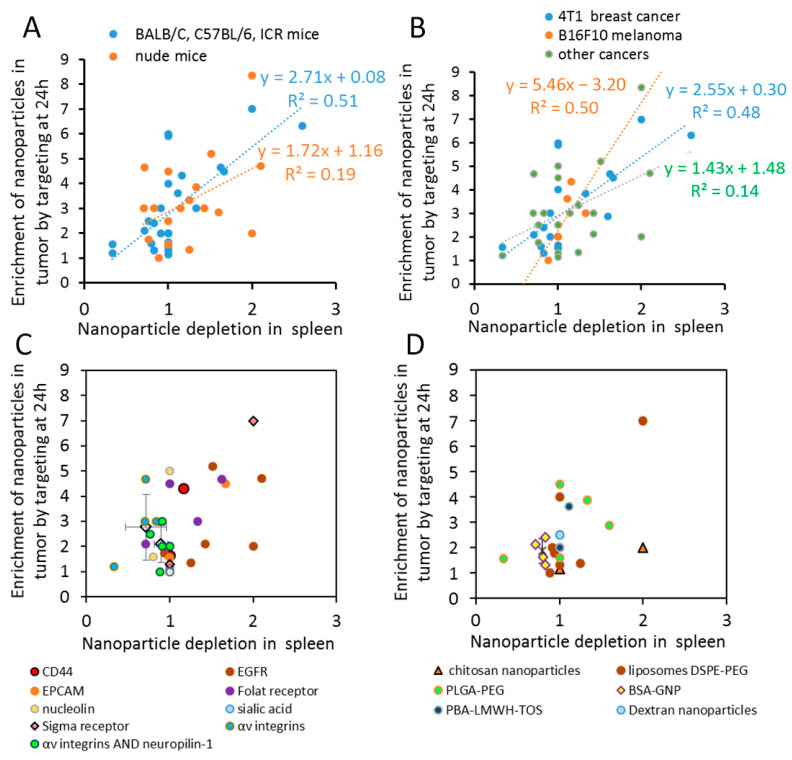
The positive correlation between accumulations of the nanoparticles in tumors and depletion in spleen is significant for the wild type mice and is more profound for integrin-targeting molecules or BSA-GNP nanoparticles. Nanoparticle *ENT* plotted vs. ratios of non-targeted to targeted nanoparticle concentrations in the spleen at 24 h after administration: (**A**) for different cancer types; (**B**) for either nude mice or mice with intact immune systems; (**C**) for targeted molecules that presented more than once in our dataset. Diamonds with error bars are averages for integrin-targeting RGD or iRGD peptides. Bars represent 95% confidence interval. (**D**) Nanoparticle types that presented more than once in our dataset. Bars represent 95% confidence interval for BSA-GNP. Notice that targeting of integrins and neuropilin-1 by iRGD peptide or applications of BSA-GNP are characterized by relatively low enrichment in tumors and accumulation in spleen, *p* < 0.05.

There was a positive correlation between nanoparticle depletion in the spleen and the accumulation in the tumors at 24 h after administration for mice with intact immune systems (Figure 2A), whereas for nude mice, the trend remained, but the significance was lost (Figure 2A). We did not observe any correlation between accumulation in tumor and liver, lung, or kidney for WT or nude mice (data not shown). Similarly, the correlations between nanoparticle accumulation in tumors and depletion in the spleen were nearly significant for 4T1 breast cancer (R = 0.49) and more significant for B16F10 melanoma (R = 0.98) while, for other tumors, the trend remained but the correlation became not significant (Figure 2B).

Further, we tried to unravel the parameters of nanoparticle targeting that determine coordinated changes in spleen and tumor accumulation. To achieve this, we selected molecules that were targeted in our dataset more than once (Figure 2C) and nanoparticle types that were used in our dataset more than once (Figure 2D). Apparently, nanoparticle targeting of integrins and neuropilin-1 by iRGD peptide generates significantly lower enrichment in tumors (*p* = 0.05) and higher accumulation in spleen (*p* = 0.014) than other nanoparticle types at 24 h after administration (Figure 2C). Similarly, it was found that BSA-GNPs accumulate in the spleen and have relatively low enrichment in tumors as a result of targeting, compared to other nanoparticles (*p* = 0.005, Figure 2D).

In Table 2, changes in the biodistribution for DSPE-PEG liposomes are sorted by the depletion of nanoparticles in the spleen by targeting molecules. In contrast to integrin targeting and BSA-GNP’s, the degree of spleen depletion for the folate receptor, CD44, and the degree of sigma receptor targeting by anizamide and EPCAM, are not consistent for different reports and display an induction of tumor accumulation and depletion in the spleen (Figure 2C). For example, for the same nanoparticle type, mice strain, and tumor type, anisamide ligand [144,145] demonstrated stronger depletion in the spleen and higher *ENT* than iRGD at 24 h after administration (Table 2).

In contrast, while some publications revealed that EGFR antibody and antibody fragments promote cellular internalization and significantly change biodistribution, inhibiting localization to the spleen and liver and inducing tumor accumulation of the nanoparticles [144,148], others reported that EGFR targeting leads to the depletion of nanoparticles in the spleen but to low accumulation in tumors [145,149,150], (Figure 2C).

We tried to estimate a contribution of the nanoparticle clearance by the spleen or liver to nanoparticle concentrations in the blood, assuming that the spleen weight is about 100–200 mg and the liver weight is 1–1.2 g for 25 g mice [151], and the blood volume is about 1.5 mL. Even 2× changes of nanoparticle accumulations in the spleen would not be sufficient to influence the blood concentrations. Moreover, we expected that the changes in the liver nanoparticle accumulation would be much more predictive than those of the spleen, given that a decrease in concentrations in the liver predicts nearly the same increase in the concentrations in blood. However, we did not observe a correlation between the changes of nanoparticle concentrations in the liver and the nanoparticle *ENT* in tumors (Figure 1B). Therefore, the mechanism is not due to the direct changes in the bioavailability of the particles.

What could cause such phenomena? When passing through the spleen, nanoparticles interact with macrophages and B cells of the white pulp, and on the venous side, they can be captured by the red pulp macrophages when passing in between endothelial cells [55]. In addition, the spleen can capture nanoparticles via marginal zone macrophages mediated by the scavenger receptors [56,57]. Notably, the marginal zone is well defined in rats and mice, whereas in humans, it is represented by the perifollicular zone, containing at least three layers [54,152,153]. It has been noted that macrophages, being the major professional nanoparticle sequestration cells, accumulate the majority of the nanoparticles in tumors [91,142]. Additionally, the spleen is a source of the tumor-associated macrophages (TAM) in the lung carcinoma model, and a splenectomy leads to a reduction in TAMs and the suppression of tumor growth [154]. During cancer rejection, the spleen and lymph nodes are the sites of cell proliferation [155]. The spleen facilitates the anti-melanoma immune response in mice [156,157] and likely in humans [158]. Moreover, in the presence of a tumor, a significant 2.6- and 4-fold decrease in particle uptake in the spleen for BALB/c and C57Bl/6 strains, respectively, was observed for 50-nm particles [63]. Altogether, this suggests that the tumor-associated immune cells travel in-between, in, and out of the spleen and tumor. One of many possible hypotheses is that the targeting molecules modulate nanoparticle sequestration via both the spleen macrophages and TAMs. Thereby, nanoparticles are going to penetrate and accumulate more deeply into the tumor tissue. Specific mechanisms of the negative correlation between nanoparticle accumulation in tumors and the spleen should be tested experimentally for each system.

Splenectomies prevent the phenomena of accelerated blood clearance (ABC) of nanoparticles [159], which is modulated by the IgM production conducted by B-cells of the marginal zone [160,161]. However, there is no evidence that the ABC develops after the use of pegylated liposomal doxorubicin in humans [162] and mice experiments demonstrated that Dox loading inhibits the ABC in mice [163]. Therefore, the biodistribution of the nanoparticles with drugs might be different upon repeated administration; however, this issue is rarely analyzed in the literature [164,165,166]. Recent data demonstrated prolonged blood circulation of the nanoparticles after administration of the anti-RBC antibodies that block nanoparticle sequestration by the mononuclear phagocyte system and a subsequent enhancement in anti-CD4 targeting and B16-melanoma xenograft treatment [64]. However, in this case, 1.5 h after administration, nanoparticles were sequestered more in the bones, lungs, and spleen, and less in the liver [64].

### 2.3. Efficient Targeting of Nanoparticle Drugs Improves Cancer Survival

**Figure 3:**
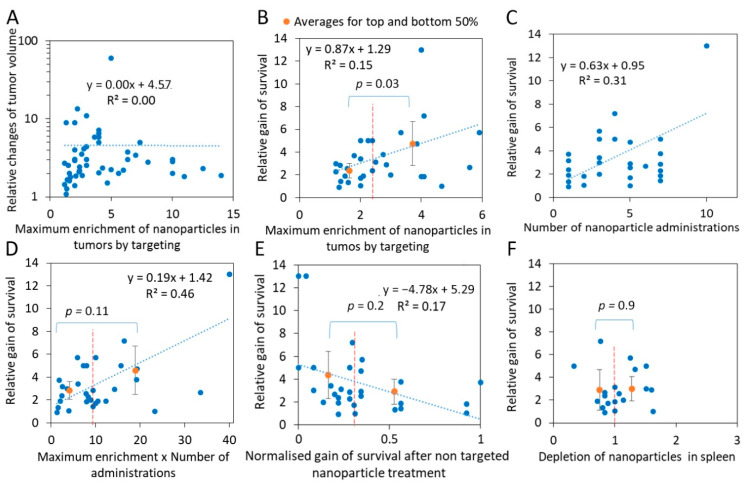
Nanoparticle targeting enhances cancer survival. Here, blue dots are raw data and orange dots are averages for top and bottom 50% of the abscissa values. (**A**) The relative gain of tumor volume (*vNT-vInitial*)/(*vT-vInitial*) does not correlate with the maximum enrichment of nanoparticle concentration in tumors by targeting. (**B**) The relative gain of survival (*sT-sContr*)/(*sNT-sContr*) correlates with the maximum *ENT*. (**C**) The gain of survival as a function of the number of drug administrations. (**D**) The gain of survival as a function of the maximum *ENT* multiplied by the number of drug administrations. (**E**) The relative gain of survival does not correlate with the normalized gain of survival for mice treated with non-targeted nanoparticles. (**F**) The relative gain of survival does not correlate with depletion of the nanoparticles in spleen.

Indeed, many examples demonstrate that the enhanced nanoparticle accumulation in tumors is translated into better cancer treatment. However, the correlation between *ENT* and relative changes in tumor volumes is not significant in the collected data (Figure 3A).

Apparently, there is a weak linear correlation between the relative gain of overall survival and the maximum enrichment of nanoparticle targeting (R = 0.15) (Figure 3B). Nonetheless, the overall relative gain of survival is higher for nanoparticles with more efficient targeting. For the top 50% vs. the bottom 50% of *ENT* values, the difference is significant, at *p* = 0.03, based on the two-tailed Student’s *t*-test. The treatment efficiency depends not only on the nanoparticle targeting per se, but equally on the tumor type, mice strain, drug type, doses, and treatment schedule, etc., which were all different in the analyzed papers. For example, tumor-treatment efficiency increased with a higher quantity of nanoparticles targeting EGFR signaling [167]. It is worth mentioning that the highest gain of the survival was achieved with the maximum number of treatments [29], and there is a weak correlation between the two (Figure 3C). The best indication would be a difference between concentrations of drugs delivered by targeted and non-targeted nanoparticles integrated over the treatment period; however, such data are rarely available. As a surrogate of such a measure, we used a cumulative enrichment measure, calculated as the number of administered treatments multiplied by the *ENT* maximum. In this case, the correlation coefficient became higher, R^2^ = 0.5, although the difference between the relative gain of survival for the top 50% vs. the bottom 50% of the cumulative enrichment became less significant *p* = 0.07 (Figure 3D). In addition, we investigated the possibility that the low efficiency of the treatment by the non-targeting nanoparticles corresponds to a higher relative gain of survival by lowering the denominator in Equation (5) (see Section 3.2). To achieve this, we compared the normalized gain of survival calculated for the non-targeted nanoparticles (Equation (6); and the relative gain of survival for targeted nanoparticles—Equation (5)). We did not observe that non-targeted nanoparticles, with either low or high effects on survival, corresponded to a high or low relative gain of survival induced by the corresponding targeting nanoparticles, although a non-significant trend was found (Figure 3E).

An analysis of 19 papers in our dataset did not reveal a correlation between the gain of cancer survival and the depletion of nanoparticles in the spleen (Figure 3F). However, more detailed investigations may reveal the role of the spleen’s sequestration of nanoparticle drugs in cancer treatment.

### 2.4. The Best Combinations of The Targeting Agent and Nanoparticle Type Are Cancer-Specific

In an attempt to analyze the factors determining efficient nanoparticle targeting, various parameters such as types of targeting molecules, nanoparticle type, size, zeta potential, dose, and circulation time were evaluated. It was reported, that targeting efficiency is lower for nanoparticles larger than 60 nm [89]. In addition, it is known that smaller particles tend to circulate for longer than larger ones [48]. Nonetheless, our analysis of the literature did not show that 200 nm particles are less efficient than 50 nm ones (Table 2, Table 3, Table 4, Table 5 and Table 6). This can be explained by the variability of the less frequently measured parameters of the nanoparticles such as the length and the density of the PEG linker that are critical for the efficiency of the targeting molecule [88]. Likewise, zeta potential, which is widely used to characterize nanoparticles, did not show any correlation with the targeting efficiency, neither does the dose (the data are not shown, see Supplementary File, Table S1). We did not find any specific nanoparticle parameter that could universally determine high *ENT*, which was not surprising given the heterogeneity of the experimental conditions. Moreover, the best combinations of the targeting agents and nanoparticle types are known to be specific for a particular tumor [7]. However, it is important to analyze efficient combinations that appear over time. Therefore, we determined the best *ENT* values across the nanoparticle and cancer types in our dataset.

First, we compared different nanoparticle types for similar targeting ligands and cancer types. Apparently, hyaluronic acid targeted liposomes (*ENT* = 6.3 [69]), outperforming solid lipid nanoparticles (*ENT* = 1.5 [168]) and less charged liposomes (*ENT* = 1.2 [169]) for the delivery of nanoparticles to the B16F10 melanoma (Table 3). In contrast, the iRGD-targeted liposomes demonstrated consistent *ENT* values in the range of 1.5–2.5 for the B16F10 [70,93,169] and B16 melanomas [103] (Table 4).

**Table 4:** Nanoparticles targeted by the iRGD peptide in the B16F10 and B16 melanomas. The data are sorted by the maximum *ENT*.

Nanoparticle Type	Targeting Molecule	Size, nm	Zeta Potential, mV	Cell Type	Tumor Type	MICE STRAIN	Maxim ENT	Reference
liposomes	iRGD HA	128	−7.4	B16F10	Melanoma xenografts	C57BL/6	2.4	[153]
liposomes DSPE-PEG	iRGD	93	−24	B16F10	Melanoma xenografts	BALB/c nude	2.0	[63]
liposomes DSPE-PEG	iRGD	90	−14.9	B16F10	Melanoma xenografts	BALB/c nude	2.0	[91]
liposomes DSPE-PEG	iRGD	95	−1.59	B16	Melanoma xenografts	C57BL/6	1.5	[96]

Then, we compared the targeting molecules and nanoparticle types for the mouse 4T1 breast cancer (Table 5).

**Table 5:** Nanoparticles targeted by different molecules in 4T1 breast cancer xenografts. The table is sorted by the maximum *ENT*.

Nanoparticle Type	TARGETING MOLECULE	Size, nm	Zeta Potent, mV	Cell Type	Tumor Type	Mice Strain	Max *ENT*	Ref.
G5-PAMAM dendrimer	IL-4Rα specific peptide	170	NA	4T1	xenografts	BALB/c	8.3	[166]
liposomes DSPE-PEG	anisamide ligand	95	40	4T1	ortotopic xenografts,	BALB/c	7.0	[146]
PECL-hyd-DOX	Folic Acid	71	NA	4T1	xenograft	BALB/c	6.3	[68]
liposomes DOTAP:DOPE	4T1 cells specific aptamer	120	35	4T1	xenografts	BALB/c	6.0	[40]
TCPP-mPEG−PLGA	NK cell membranes	85	−11.8	4T1	xenografts	BALB/C	5.9	[170]
liposomes DSPE-PEG-	Peptides c(RGDfC), P-selectin, CREKA, EGFR	100	3	4T1	lung metastasis of ortotopic xenografts	BALB/c	5.6	[171]
silver-coated gold nanorods	EpCam Ab	36	NA	4T1	orthotropic xenografts	BALB/c	4.5	[172]
liposomes DSPE-PEG	nRGD (modified iRGD)	152	−13.6	4T1	xenografts	BALB/c	4.0	[72]
PLGA-PEG	neovessels-targetable K237 peptide and Ep23 aptamer	122	−25	4T1	orthotropic xenografts	BALB/c nude	3.9	[39]
liposomes DSPE-PEG-DBCO/ PLGA	iRGD	112	−34.1	4T1	orthotropic xenografts	BALB/c	3.0	[101]
Fe3O4 nanoparticles	amino-terminal fragment of urokinase plasminogen activator	18	−11	4T1	xenografts (also metastasis)	BALB/c nude	3.0	[173]
PLGA-PEG	K237 peptide	122	−28	4T1	orthotropic xenografts	BALB/c nude	2.9	[39]
BSA-GNP	glutamine	13	NA	4T1	orthotropic xenografts	BALB/c	2.4	[174]
BSA-GNP	Folic Acid	13	NA	4T1	orthotropic xenografts	BALB/c	2.1	[174]
RD NPs connected to GNPs in a manner comparable to satellites	RDGfK	130	−6	4T1	xenografts	BALB/c	2.0	[175]
liposomes DSPE-PEG	iRGD	115	−34	4T1	xenografts	BALB/c	2.0	[102]
liposomes DSPE-PEG	iRGD	166	−11.4	4T1	xenografts	BALB/c	2.0	[72]
graphene PEG conjugates	CD105	27	−0.8	4T1	xenografts	BALB/c	1.9	[176]
Keratin-HA gels	HA	80	−13	4T1	xenografts	BALB/c	1.7	[61]
BSA-GNP	AS1411 aptamer	15.2	NA	4T1	xenografts	BALB/c	1.6	[37]
PLGA-PEG	Ep23 aptamer	122	−29	4T1	orthotropic xenografts	BALB/c nude	1.6	[39]
PLGA-PEG	malamide, non/spec plasma proteins	175	−11.6	4T1	xenografts	BALB/c	1.6	[177]
HA-PTX MATT b-casein	HA	234	−8.5	4T1	xenografts	BALB/c	1.4	[178]
BSA-GNP	glucose	13	NA	4T1	orthotropic xenografts	BALB/c	1.3	[174]

The 4T1 mouse breast cancer was targeted the most effectively by the IL-4Rα specific peptide conjugated to the G5-PAMAM dendrimer (*ENT* = 8.3) [166] and by the sigma receptor-specific anisamide ligand coupled to the DSPE-PEG liposomes (*ENT* = 7) [146] (Table 5).

The iRGD peptide is widely investigated in the nanomedical field and is already in clinical trials. Therefore, we determined the conditions in which the iRGD performed the best. The greatest result, *ENT* = 10, was demonstrated in the original papers for the iRGD-targeted liposomes for human 22R1 prostate cancer and for BT474 breast cancer xenografts in nude mice [31,100] (Table 6).

**Table 6:** Nanoparticles targeted by the iRGD peptide in prostate, breast, and glioma cancers. The data are sorted by the maximum *ENT*.

Nanoparticle Type	Targeting Molecule	Size, nm	Zeta Potent, mV	Cell Type	Tumor Type	Mice Strain	Max *ENT*	Ref.
Liposome	iRGD	NA	NA	22Rv1	Prostate orthotopic	nude	14	[100]
Liposome	iRGD	NA	NA	22Rv1	Prostate orthotopic	nude	14	[100]
BSA (Abr)	iRGD	120	NA	BT474	Breast	nude	12.5	[31]
BSA (Abr)	iRGD	120	NA	BT474	Breast	nude	11	[100]
BSA (Abr)	iRGD	120	NA	22Rv1	Prostate orthotropic	nude	10	[31]
BSA (Abr)	iRGD	120	NA	22Rv1	Prostate orthotropic	nude	8	[60]
PE- PAMAM dendrimer	iRGD	20	2.45	C6	Glioma Intracranial	ICR	4.1	[101]
PLGA/liposomes DSPE-PEG-DBCO	iRGD	112	−34.1	4T1	Breast orthotropic	BALB/C	3	[92]
exosomes	iRGD	97	NA	MDA-MB-231	Breast	BALB/c nude	3	[31]
Fe3O4 nanoworms	iRGD	85	NA	22Rv1	Prostate orthotropic	nude	2	[102]
liposomes DSPE-PEG	iRGD	115	−34	4T1	Breast	BALB/C	2	[72]

However, for cancers in mice, the best *ENT* value of 3 for the iRGD was achieved for PLGA/DSPE-PEG-DBCO liposomes for 4T1 breast cancers [101] and *ENT* = 2.4 was achieved for DPPE liposomes for B16F10 melanoma (Table 4 and Table 6) [169]. A relatively high *ENT* of 4.1 was achieved for the iRGD-targeted PEGylated polyamidoamine (PAMAM) dendrimers for the rat intracranial glioma [60]. Thereby, we determined the best targeting agent and nanoparticle type combinations for the specific cancer models.

### 2.5. Combinatorial Targeting Increases Nanoparticle Accumulation in Tumors

Tumors are characterized by combinations of molecules overexpressed in the endothelium, cancer cells, and stromal tissue with a high concentration of the secreted molecules that are specifically associated with immunosuppressive microenvironments, such as TGF-b or IF2, or by high concentrations of the low molecular weight hydrogen, lactate, and adenosine [179]. Therefore, an attractive idea is to target several molecules, or ultimately, create nanoparticles to perform logical operations that could be highly sensitive to such combinations of cancer-specific molecules [16,129,180,181,182,183,184,185].

This approach has led to the combinatorial targeting of the low pH of the tumor microenvironment and the overexpression of the sialic acid residues by cancer cells using the pH-sensitive “Fructose-Blockage” of phenylboronic acid [181]. This enhanced accumulation of the nanoparticles to *ENT* = 3.62 in comparison with *ENT* = 2 for the phenylboronic acid only for B16F10 melanoma, and reduced accumulation in normal tissues [181]. Similarly, the pH-sensitive mannose, PEGylated with an acid-sensitive PEG amphiphile, the PEG-hydrazone-C18 prevented accumulation of the nanoparticles in the liver (most likely due to interactions with the M2 liver-resident macrophages) and enhanced the targeting of tumor-associated macrophages in the acidic microenvironment of the B16F10 melanoma, reaching *ENT* = 4, in comparison to *ENT* = 1.2 for unblocked mannose [129].

**Figure 4:**
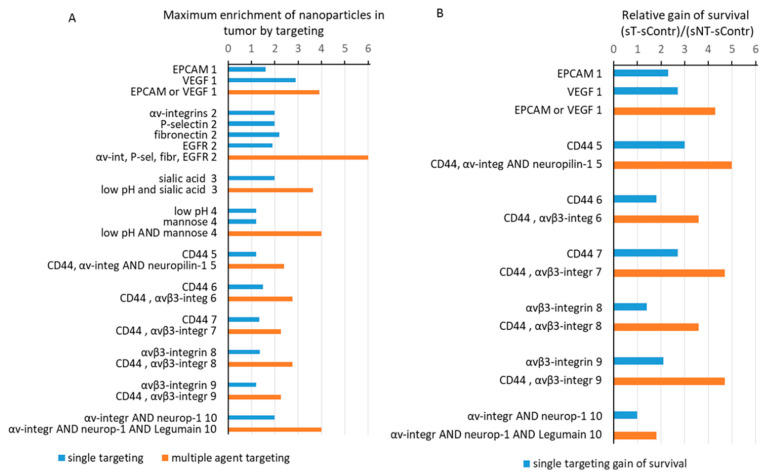
Combinations of targeting ligands result in higher targeting enrichment and better survival after treatment. (**A**) *ENT* values for single-ligand targeted nanoparticles are in blue, and for combinatorial targeting, they are shown in orange; (**B**) relative gains of survival for single-ligand targeted nanoparticles are in blue and those observed for combinatorial targeting are in orange. An index shown next to the labels facilitates separation of the groups.

The creation of artificial signaling cascades, best exemplified by the iRGD [31], or the use of naturally occurring signal amplification cascades such as blood coagulation [181,186] produced strong induction of the nanoparticle concentrations in tumors with the increasing of the therapeutic efficiency. Other examples include the utilization of heat or nanoparticle-induced blood coagulation cascades to modulate nanoparticle localization [182] or the targeting of radiation-induced *p*-selectin expression [183]. Parallel targeting of several molecules results in a higher concentration of nanoparticles in tumors (*p* = 3 × 10^−6^, Student’s *t*-test) and prolonged survival (*p* = 2 × 10^−5^, Student’s *t*-test) (Figure 4).

This can be exemplified by the four peptides targeting αβ-integrins—c(RGDfC), the P-selectin-binding peptide CDAEWVDVS, the CREKA peptide with high affinity to fibronectin, and the EGFR-selective peptide CYHWYGYTPQNV [171]. CD44 targeting using hyaluronic acid-modified liposomes co-administered with the tumor-penetrating peptide-iRGD produced *ENT* values of 2.4 and 1.2 for the hyaluronic acid only [169]. Similarly, CD44 targeting by hyaluronic acid together with integrin targeting by tetraiodothyroacetic acid produced an *ENT* of 2.75, whereas the hyaluronic acid only produced an *ENT* of 1.5 and tetraiodothyroacetic acid yielded an *ENT* of 1.35 [168]. Another interesting example is a substantial induction of the survival and inhibition of the tumor growth by the nanoparticles targeted by the nRGD-modification of the tumor-penetrating peptide iRGD, with the AAN peptide extension recognized by the legumain-lysosomal cysteine protease, which is overexpressed in tumor cells and tumor-associated macrophages [72].

### 2.6. Cases with the Highest Cancer Survival Gain after a Targeted Nanoparticle Treatment

The highest reported 13× relative gain of survival was obtained for MC38 colon cancer xenografts C57BL/6 mice model treated with PLGA-PEG nanoparticles that specifically target T-lymphocytes via the F(ab’)2 fragment of the anti-PD-1 antibodies with *ENT* = 4 [29]. The caveat of our analysis applied to this paper is that *ENT* was measured using the B16 melanoma model, whereas the therapeutic efficiency studies were conducted on the MC38-derived cancers. The authors explained the rationale behind this experimental design: “MC38 was favored over B16 for in vivo studies because the latter are not greatly affected by anti-PD-1 monotherapy” [187]. Nonetheless, it was the T-cells that were targeted, not cancer cells; therefore, we decided to include these data. The drug used in this study was SD-208—an inhibitor of TGFβRI kinase [188]—that blocked immunosuppressive pathways induced by the TGFβ, which is frequently overexpressed in tumor tissue. Another drug that they used and which produced the second-best results was the Toll-like receptor (TLR) 7/8 agonist R848 (resiquimod) [189].

The next highest survival gain of 7× was achieved in the C6 intracranial glioma of ICR mice treated by the PE-PAMAM dendrimer loaded with doxycycline [60] and av-integrins and neuropilin-1 targeted by the iRGD peptide. The *ENT* maximum was 4.1, similar to the previous case. This was followed by a survival gain of 5.7× reported for the intracranial glioma model generated from the C6 cells that were treated with PG-PCL nanoparticles delivering paclitaxel (PTX), targeted by the composite peptide (Maximum *ENT* = 3.3) with affinity to both PD-L1 and surface heparan sulfate polysaccharides, which were upregulated in the tumor vasculature [190]. 

The third highest survival gain of 5× was observed for the melanoma metastasis model, in which B16F10 cells were injected into the tail of C57BL/6 mice, treated with liposomes that delivered Dox, and targeted both CD44 and integrin αvβ3 via the combination of hyaluronic and tetraiodothyroacetic acids, with an *ENT* of 2.3 [168]. B16F10 xenograft mice were also successfully treated in this study [168].

Another case with 5× survival gain was observed for the melanoma xenograft model, wherein B16F10 cells were injected into the flanks of C57BL/6 mice, treated with the liposomes that delivered PTX, and targeted both CD44, neutrophilin-1, and integrin αvβ3 with an *ENT* of 2.4 achieved by the coadministration of the iRGD peptide with hyaluronic acid-modified liposomes [169]. A similar case, with a survival gain of 5x, was discussed for the intracranial glioma C6 model with cells injected into nude mice, which were treated with the PAMAM dendrimer nanoparticles delivering Dox, which targeted αvβ3 integrin via the RGD peptide, with an *ENT* of 2.0 [33].

A significant 4.7× gain of survival was also achieved for 4T1 breast cancer orthotropic xenograft in the BALB/C mice treated with the PLGA-PEG nanoparticles delivering paclitaxel, with an *ENT* of 3.9 achieved by targeting of EPCAM with the Ep23 aptamer and the targeting of VEGFR with the K237 peptide [39]. Remarkably, an idea that arose from the study was to target circulating tumor cells that detach from the primary tumor site and act as ‘seeds’ for metastasis. They used in vivo flow cytometry to detect interactions between intravenously injected 4T1-GFP cells and DiD-labeled nanoparticles. Moreover, they detected the binding of nanoparticles to 4T1-GFP cells that homed into the lungs 4 h post-injection [39].

The 2.7× gain of survival for the highest *ENT* = 5.8 value in the group with survival data (Figure 3B) was achieved with the hyaluronic acid targeted liposomes, wherein paclitaxel was delivered to the B16F10-CD44+ stem-like cells injected into the tail veins of the C57BL/6 mice to create a murine lung metastasis model [71].

Altogether, the collected data demonstrate that the use of agents that increase the concentration of therapeutic nanoparticles in tumors is a valuable strategy to improve cancer survival.

## 3. Materials and Methods

### 3.1. Search Strategy

**Figure 5:**
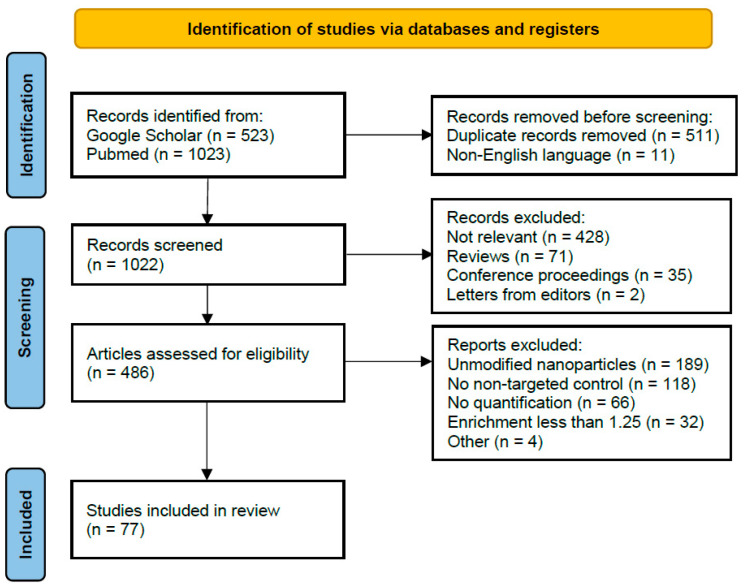
PRISMA flow diagram for systematic review depicting phases of identification of studies.

The literature was searched for biodistribution studies of ligand-targeted nanoparticles in general that specifically quantified the biodistribution of the nanoparticles. The timeframe of the search included all studies until the end of December 2020. Google Scholar and Pubmed were used with search terms such as “targeted delivery”, or “nanoparticles biodistribution”, “biodistribution metallic/polymeric/organic/etc. nanoparticles/nanomaterials” in all variations. Any potentially relevant meeting abstracts and articles found in their reference lists were reviewed and considered for inclusion according to the flowchart shown in Figure 5. After preliminary screening of abstracts, papers were subjected to evaluation according to the following criteria: Firstly, articles without quantification of the biodistribution parameters were omitted. Secondly, publications that did not report tumor accumulation of non-targeted nanoparticles were excluded. Then, we excluded publications that used targeting therapy such as BRAF or MEK inhibitors, but not targeted nanoparticles per se. Lastly, we analyzed only studies demonstrating targeting-induced enrichment of the nanoparticles in tumors of 1.25 times or higher. For biodistribution analysis, we excluded publications in which the concentration of the best targeting nanoparticles in the tumor did not exceed 0.15 of that in the spleen or liver (*n* = 2). In addition, a number of articles were excluded that turned out to be unusable due to lack of information during a detailed examination.

**Figure 6:**
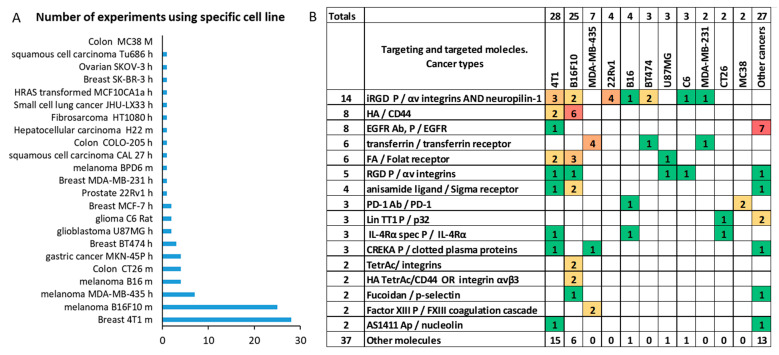
Dataset characteristics. (**A**) Number of experiments performed using different cell lines from either human or mouse; (**B**) Number of experiments for the top 16 most frequently annotated targeting molecules together with targeted molecules and cancers. The number of extracted datasets is highlighted by coloring. The remaining 37 targeting and targeted molecule combinations were tested only once. Here, *P* stands for peptide, *Ac* is acid, *Lig* is a ligand, *Ab* is an antibody, *Ap* is an aptamer.

Data were collected from studies that used the same targeting molecules but in different settings such as various nanoparticle types or cancer models such as human melanoma and breast cancers as well as prostate, glioblastoma, colon, and other cancers. In addition, the papers were analyzed that utilized a cancer-specific sequence of events [31] or natural signaling cascades such as blood coagulation [182], or radiation-induced p-selectin expression [183], among others. The summary of the dataset is presented in Figure 6, Table 2, Table 3, Table 4 and Table 5 and is available in the Electronic Supplementary File Table S1. 

### 3.2. Data Analysis

To compare non-targeted and targeted nanoparticles, the enrichment of nanoparticles in a tumor by targeting (*ENT*) was calculated as:

*ENT = cT/cNT*, (1)

where *cT* is the measured concentration of targeted nanoparticles in tumors, *cNT* is the concentration of non-targeted nanoparticles in tumors. The *cT* and *cNT* values were quantified from the graphs or tables presented in corresponding papers. In the cases where the authors did not quantify images, we did our best to estimate the average intensity reflecting the nanoparticle concentration in tumors using ImageJ v. 1.8.0 software (Kensington, MD, USA). The *ENT*’s obtained at different time points were tabulated and the maximum of the data was calculated. For papers comparing several ligands for nanoparticle targeting, all agents in the paper were included [72,89,168,171,174]. The *ENT* values in our dataset ranged from 1.3 to 30. The vast majority of biodistribution data were collected at the 24 h time point or at the latest time since the nanoparticle injection.

To characterize the effect of the nanoparticle targeting on organ sequestration, for each organ we calculated the depletion ratio (*DR*):

*DR = cNT_o_/cT_o_*, (2)

where *cNT_o_* is the concentration of non-targeted nanoparticles in the organ, *cT_o_* is the concentration of targeted nanoparticles in the organ.

Relative changes of tumor volumes (*TV*) for mice treated with targeted vs. non-targeted nanoparticles were calculated as follows:

*TV = vNT/vT*, (3)

where *vT* is the last measured tumor volume treated with targeted nanoparticles, *vNT* is the last measured tumor volume treated with non-targeted nanoparticles, and *VInit* is the tumor volume at the start of the treatment.

Relative gains of survival (*SG*) of mice treated with targeted vs. non-targeted nanoparticles were calculated as follows:

*SG = (sT − sContr)/(sNT − sContr)*, (4)

where *sT* is median survival for mice treated with cancer-targeted nanoparticles, *sNT* is median survival for mice treated with non-targeted anticancer nanoparticles, and *sContr* is median survival for untreated or PBS-treated mice.

For two cases [29,33], *sNT* survival was equal to controls, and the relative gains of survival were calculated as follows:

*SG = (sT − sContr)*/1, (5)

To characterize the effect of non-targeted nanoparticles on the relative gain of survival, normalized gains of survival (*nSG*) were calculated as follows:

*nSG = (sNT − sContr)/sContr)*, (6)

Statistical significance was calculated in Microsoft 365 Excel (Redmond, WA, USA) using a two-tailed Student’s *t*-test for non-equal standard deviations. Bars represent 95% mean confidence intervals. Regression and correlation coefficients were calculated using standard Excel tools.

## 4. Conclusions

Our analysis revealed that an increase in nanoparticle concentrations in tumors via the targeting of molecules positively correlates with the reduction in nanoparticle concentrations in the spleen, but not in the liver, lung, kidney or heart. We found that αβ-integrin targeting by RGD or iRGD peptides increases—whereas nanoparticle targeting by anisamide, folic acid or hyaluronic acid might decrease—accumulation in the spleen. The correlation between accumulation of the nanoparticles in the spleen and the tumor was evident when the breast cancer or melanoma wild type mice were filtered, suggesting that the phenomena is likely dependent on the type of cancer. A hypothetical mechanism could be that targeting molecules modulate nanoparticle sequestration by splenic and tumor macrophages, leading to deeper penetration of the nanoparticles in the tissue and accumulation in tumors. Experimental studies are needed to determine the origin and significance of the correlation between tumor and splenic nanoparticle accumulation. The medial survival in mice models is increasing with the induction of the nanoparticle concentrations by the targeting molecules. However, we did not find that treatment efficiency was increased with the decreasing of the nanoparticle splenic accumulation. Based on the analyzed data, it was found that neither hydrodynamic radius variation from 50 to 200 nm nor the zeta potential showed any correlation with the targeting efficiency, which was mainly correlated with the targeting molecule and animal model used. It is important to note that the heterogeneity of the research approaches and data representation in the field of anti-cancer nanomedicine complicates the analysis of the results and the determination of general features and elicitation of structure–activity relationships. Finally, the combinations of molecules for the targeting of therapeutic nanoparticles result in higher nanoparticle accumulation in tumors and improve cancer survival.

## Figures and Tables

**Table 1:** Active targeting strategies currently in the clinical trials (https://clinicaltrials.gov, accessed on 25 November 2020).

ID	Title	Targeting Molecule	Nanoparticle
NCT02369198	MesomiR 1: A Phase I Study of TargomiRs as 2nd or 3rd Line Treatment for Patients With Recurrent MPM and NSCLC	Anti-EGFR bispecific antibody	Buds of bacterial cytoplasm
NCT02106598	Anti Targeted Silica Nanoparticles for Real-Time Image-Guided Intraoperative Mapping of Nodal Metastases	Integrin-binding cRGDY peptide	Silica nanoparticles
NCT01702129	EGFR Immunoliposomes in Solid Tumors	EGFR antibody	Liposomes
NCT00505713	Safety and Efficacy Study Using Rexin-G for Sarcoma	Collagen-binding viral envelope peptide	Retroviral
NCT00505271	Safety and Efficacy Study Using Rexin-G for Breast Cancer	Collagen-binding viral envelope peptide	Retroviral
NCT02354547	Phase II Study of Combined Temozolomide and SGT-53 for Treatment of Recurrent Glioblastoma	Anti-transferrin scFv antibody fragment	Liposomes
NCT02766699	A Study to Evaluate the Safety, Tolerability, and Immunogenicity of EGFR(V)-EDV-Dox in Subjects With Recurrent Glioblastoma Multiforme	Anti-EGFR bispecific antibody	Buds of bacterial cytoplasm
NCT03517176	CEND-1 in Combination With Nab-paclitaxel and Gemcitabine in Metastatic Pancreatic Cancer	αv-integrins targeted and neuropilin-1 mediated tumor-penetrating iRGD peptide	Co-administration with nab-paclitaxel

**Table 2:** Changes in the biodistribution for DSPE-PEG liposomes are sorted by the depletion of nanoparticles in the spleen by targeting molecules.

Nanoparticle Type	Targeting Molecules/Aims	Size, nm	Zeta Potent, mV	Cell Type	Tumor Type/Strain	*ENT* at 24 h	Depletion in				Ref.
							Liver	Spleen	Lung	Kidney	
liposomes DSPE-PEG	anisamide lig/ Sigma-R	95	40	4T1	ortotopic xenogr BALB/C	7	1.7	2.0	1.7	1.0	[146]
liposomes DSPE-PEG	anisamide lig/ Sigma-R	145		BPD6	xenogr C57BL/6	1.3	1.0	1.0	1.4	3.3	[147]
liposomes DSPE-PEG	iRGD/ av-integr neirophil-1	166	−11.4	4T1	xenogr BALB/C	2.0	1.4	1.0	1.1	1.2	[72]
liposomes DSPE-PEG	nRGD/ av-integr neirophil-1 Legumain	152	−13.6	4T1	xenogr BALB/C	4.0	1.4	1.0	1.6	1.2	[72]
liposomes DSPE-PEG	iRGD/ av-integr neirophil-1	115	−34	4T1	xenogr BALB/C	2	1.0	0.9	0.7	1.0	[102]
liposomes DSPE-PEG	iRGD/ av-integr neirophil-1	93	−24	B16F10	xenogr BALB/c nude	1	0.7	0.9	1.1		[70]

**Table 3:** Nanoparticles targeted by the HA in the B16F10 melanoma xenografts. The data are sorted by the maximum *ENT*.

Nanoparticle Type	Targeting Molecule	Size, nm	Zeta Potent, mV	Cell Type	Tumor Type	Mice Strain	Max *ENT*	Ref.
liposome	HA	190	−22.7	B16F10	Melanoma xenografts	C57BL/6	6.3	[69]
solid lipid	HA	190	32	B16F10 CD44+	Melanoma metastasis	C57BL/6	5.6	[71]
cationic BSA-based	HA	180	30	B16F10	Melanoma metastasis	C57BL/6	3	[124]
solid lipid	HA	225	40	B16F10	Melanoma xenografts	C57BL/6	1.5	[168]
solid lipid	HA	225	40	B16F10	Melanoma metastasis	C57BL/6	1.3	[168]
liposome	HA	128	−7.4	B16F10	Melanoma xenografts	C57BL/6	1.2	[169]

## Supplementary Materials

The following are available online at https://www.mdpi.com/article/10.3390/ijms222313011/s1.
